# *Osterix-Cre* Labeled Progenitor Cells Contribute to the Formation and Maintenance of the Bone Marrow Stroma

**DOI:** 10.1371/journal.pone.0071318

**Published:** 2013-08-08

**Authors:** Yaling Liu, Sara Strecker, Liping Wang, Mark S. Kronenberg, Wen Wang, David W. Rowe, Peter Maye

**Affiliations:** Department of Reconstructive Sciences, University of Connecticut Health Center, Farmington, Connecticut, United States of America; Rutgers - New Jersey Medical School, United States of America

## Abstract

We have carried out fate mapping studies using *Osterix*-EGFPCre and *Osterix*-CreERt animal models and found Cre reporter expression in many different cell types that make up the bone marrow stroma. Constitutive fate mapping resulted in the labeling of different cellular components located throughout the bone marrow, whereas temporal fate mapping at E14.5 resulted in the labeling of cells within a region of the bone marrow. The identity of cell types marked by constitutive and temporal fate mapping included osteoblasts, adipocytes, vascular smooth muscle, perineural, and stromal cells. Prolonged tracing of embryonic precursors labeled at E14.5dpc revealed the continued existence of their progeny up to 10 months of age, suggesting that fate mapped, labeled embryonic precursors gave rise to long lived bone marrow progenitor cells. To provide further evidence for the marking of bone marrow progenitors, bone marrow cultures derived from *Osterix*-EGFPCre/*Ai9* mice showed that stromal cells retained Cre reporter expression and yielded a FACS sorted population that was able to differentiate into osteoblasts, adipocytes, and chondrocytes *in vitro* and into osteoblasts, adipocytes, and perivascular stromal cells after transplantation. Collectively, our studies reveal the developmental process by which *Osterix-Cre* labeled embryonic progenitors give rise to adult bone marrow progenitors which establish and maintain the bone marrow stroma.

## Introduction

The bone marrow contains many non-hematopoietic cell types that have been collectively referred to as the stroma. Known cell types within the stroma include: (1) osteoblasts, which enclose the marrow compartment in bone tissue, (2) endothelial and smooth muscle cells, which are organized into a complex vascular network composed of arterioles, capillaries, sinusoids, and a large central vein, (3) sensory and sympathetic nerve fibers, glia, and perineural cells that innervate the marrow compartment to form a neural network, (4) adipocytes, that may support metabolic functions of the bone marrow and (5) stromal cells, which support hematopoiesis and retain skeletal potential.

The developmental origin(s) of cell types that comprise the bone marrow stroma including their resident progenitor cell populations remains poorly understood. *Ex vivo* studies provided evidence that cells derived from the perichondrium migrate into the bone marrow cavity during its formation and not only contribute to cells of the osteoblast lineage, but also transiently contributed to endothelial cells within the bone marrow vasculature [1]. This latter work was supported by genetic fate mapping studies using a temporally controlled *Osterix*-CreERt (*ORt*) animal model. Tracing of Osterix labeled embryonic perichondrial cells, referred to as osteoprecursors, revealed their ability to migrate into the bone marrow during its formation and contribute to trabecular bone formation [2]. Interestingly, ORt fate mapping also showed the likely marking of bone marrow stromal cells [2].

Our knowledge of how different cellular components of the stroma are maintained during adolescent and adult life remains limited. However, the ability to harvest multipotent stromal cells from the adult bone marrow of a variety of animal species has contributed to the general belief that the bone marrow contains adult stem cells likely to be involved in the maintenance of the stroma. Work by Friedenstein led to the initial discovery that the bone marrow contained multipotent stromal cells that retained skeletal potential and supported a hematopoietic environment [3], [4], [5], [6]. Thereafter, several groups have confirmed Friedenstein’s work and have more extensively characterized what is now most commonly referred to as bone marrow derived mesenchymal stem cells (BMSCs) [7], [8], [9], [10], [11], [12], [13], [14], [15], [16], [17].

The identity of non-hematopoietic, adult stem cell populations within the bone has increasingly become a topic of great interest. Work by Sacchetti et al. were the first to provide evidence that multipotent stromal cells were often associated with the bone marrow perivasculature. In humans, CD146+ bone marrow cells identified an adventitial reticular cell population that displayed multipotent BMSC-like properties [17]. Later studies in mice provided further support for the perivascular identity of multipotent stromal cells using *Cxcl12-EGFP* and *Nestin-EGFP* reporter mice, in which labels are expressed in bone marrow perivascular cells that also display BMSC-like properties [13], [18]. Interestingly, gene expression analyses of CD146, Cxcl12, and Nestin isolated bone marrow cells suggested they may all identify a similar perivascular cell population based on their common expression of *Cxcl12*, *Stem Cell Factor*, and *Angiopoietin 1* [13], [17], [18].

In this study, we have investigated the historical marking of bone marrow cell types labeled using two different Osterix-Cre animal models. Similar to a previous study, we show that *Osterix*-Cre fate mapping does mark bone marrow stromal cells. However, broader examination of the stroma reveals a much greater repertoire of cell lineages labeled by Osterix-Cre fate mapping. Both constitutive and temporal Osterix-Cre fate mapping resulted in the labeling of a variety of bone marrow cell lineages including osteoblast, adipocyte, smooth muscle, perineural, and stromal. Notably, prolonged embryonic temporal fate mapping showed that the progeny of *Osterix-*Cre marked embryonic precursors persisted in the bone marrow, potentially revealing the labeling of adult progenitor cells that are responsible for maintaining the stroma. Further characterization of stromal cells labeled by Osterix-Cre fate mapping reveals their multi-lineage potential as a population, substantiating the marking of bone marrow progenitor cells. We discuss the implications of this data with regard to understanding bone growth, the marrow environment, and bone marrow progenitor cells.

## Materials and Methods

### Animals

Mouse lines were obtained from: *Osterix*-EGFPCre (Jackson Laboratories, Stock Number: 006361), *Osterix*-CreERt (generously provided by Dr. Henry Kronenberg), *Ai9* (Jackson Laboratories; Stock Number: 007909) and NIHIII (Charles River; Strain Codes 201 and 202). This study was carried out in strict accordance with the recommendations in the Guide for the Care and Use of Laboratory Animals of the National Institutes of Health. The Animal Care Committee of the University of Connecticut Health Center approved protocols 2010-605, 100515-0915, and 100547-1015 for this study. All surgery was performed under ketamine/xylazine anesthesia, and all efforts were made to minimize discomfort.

### Harvesting and Cell Culture of Stromal Cells from Mouse Bone

Offspring from *Osterix*-EGFPCre/*Ai9* matings were used to derive BMSC cultures. In brief, 2- to 4-month-old mice were sacrificed by CO2 asphyxiation followed by cervical dislocation. Femurs and tibia were dissected from the surrounding tissues. The epiphyseal growth plates were removed and the bone marrow was collected by flushing with αMEM culture medium containing 100 U/ml penicillin, 100 µg/ml streptomycin and 10% FCS (Hyclone) with a 27 gauge needle. Single cell suspensions were prepared by gently mixing the cells with a pipette followed by filtration through a 70-µm strainer. Cells were centrifuged at 350 g for 10 min and plated at a density of 1.2 × 10?6 cells/cm2. At day 4, the media was changed. On day 5, the cells were sorted and processed.

### In Vitro Differentiation of Stromal Cells

OC9+ and OC9− cells were FACS isolated from day 5 stromal cultures. Cells were plated as confluent spots for differentiation as previously described [19], [20]. For osteogenic differentiation cells were grown in αMEM medium with 10% FCS, 100 μ/ml penicillin, 100 µg/ml streptomycin, 50 µg/ml ascorbic acid, and 8 mM 2-glycerol phosphate. For adipogenic differentiation cells were cultured in 10% FCS in αMEM medium containing 1.0 µM insulin and 0.5 µM rosiglitazone. For chondrogenic differentiation cells were cultured in high-glucose DMEM supplemented with ITS+1, 50 µg/ml ascorbic acid, 100 µg/ml sodium pyruvate, 0.1 µM dexamethasone, 100 units/100 µg penicillin/streptomycin, 40 µg/ml L-proline and 10 ng/ml TGF-β3. All *in vitro* differentiation experiments were biologically replicated three times with comparable outcomes.

Cultures were harvested at progressive time points of differentiation for quantitative RT-PCR. Terminally differentiated cultures were also assayed for osteogenesis by von Kossa staining, adipogenesis by Oil Red-O, and chondrogenesis by Alcian blue as previously described [19], [20].

### Quantitative RT-PCR

RNA purification was carried out according to manufacturer’s recommendations (Macherey- Nagel). cDNA was prepared from 1 ug of RNA/sample using the SuperScript II Reverse Transcriptase kit (Life Technologies). QPCR was carried out using SybrGreen PCR Master Mix (Qiagen) in an ABI 7900HT (Applied Biosystems). PCR primer sequences for gene expression analyses are listed in Table S1.

### Dissection, Embedding, and Cryohistology

Tissues were dissected and fixed in 10% phosphate-buffered Formalin for 4 days at 4°C. Bone tissues from two week old or older mice were decalcified in 14%EDTA for 4–7 days, depending on animal age. Tissues were then placed in 30% sucrose overnight and embedded in Cryomedia (Thermo Scientific) as previously described [21]. Frozen 7 µm sections were collected using a Cryofilm type II tape transfer system (Section-Lab) in a Leica Cryostat. Sections were mounted in a 50% PBS-buffered glycerol solution for imaging.

### Immunostaining of Tissue Sections

Immunostaining was carried out using standard methodologies. Primary antibodies were diluted in 10% blocking serum (Osterix; diluted 1∶2000, Santa Cruz Biotechnologies, CD31; diluted 1∶20, R&D Systems, CD106, diluted 1∶100, R&D Systems, Smooth Muscle Alpha Actin; diluted 1∶250, Sigma-Aldrich, Collagen IV, diluted 1∶500, Abcam, Perilipin A, diluted 1∶500, Abcam, Neurofilament M, diluted 1∶500, EMD Millipore) and incubated either overnight at 4°C or for two hours at room temperature. After incubation, sections were gently washed three times, fifteen minutes per wash, in PBS. Secondary antibodies were then diluted in blocking serum (goat anti-rabbit Alexa Fluor 488, diluted 1∶500, Life Technologies, donkey anti-goat Dylight 488, diluted 1∶200, Jackson Immunochemicals) and incubated on tissue sections for two hours at room temperature. Finally, tissue sections were washed three times, for fifteen minutes each, in PBS, mounted with 50% glycerol in PBS and imaged with a fluorescence microscope (Zeiss, Observer Z.1).

### Induction of Cre Recombinase Activity with Tamoxifen

To examine the functionality of *Osterix*-CreERt mice, 1 month old *Osterix*-CreERt/+;*Ai9*/+ dual transgenic animals (n = 2/treatment) were injected with tamoxifen (Sigma; 0.025 and 0.05 mg tamoxifen/g weight of animal) or vehicle (corn oil). Tamoxifen (0.05 mg/g weight of animal) was also injected into *Osterix*-CreERt/+ (n = 2) and Ai9/Ai9 (n = 2) mice, which were used as negative controls. Examination of Cre reporter expression in whole mount and tissue sections verified the non-leaky nature of vehicle-treated *Osterix*-CreERt mice up to 1 month of age. For embryonic fate mapping, timed pregnancies were carried out for *Osterix*-CreERt and *Ai9* Cre reporter intercrosses. At 14.5dpc, 0.05 mg tamoxifen/g weight of animal was I.P. injected into pregnant females (n = 6). Offspring were sacrificed at 15.5dpc (n = 3+), 1 week (n = 3), 4 weeks (n = 3), 8 weeks (n = 3), 7 months (n = 3) and 10 months (n = 3) of age for histological examination and immunostaining.

### FACS Isolation of OC9+ Stromal Cells

Bone marrow derived stromal cultures were grown for 5 days and sorted using a FACS Aria II (BD) by the UCONN Flow Cytometry Core. Prior to sorting, adherent cells were washed twice with PBS and digested with a mixture of 0.1% Collagenase P (Roche), 0.1% Hyaluronidase (Sigma), 2% FBS (Hyclone), 49% OPTI-MEM (Gibco), and 49% PBS (Gibco) for 10minutes at 37°C, scraped, then digested for an additional 5 minutes. Cells were then resuspended in media containing 2% FBS, 49% OPTI-MEM, and 49% PBS for sorting. Cells were sorted for tdTomato using a yellow-green laser line (561 nm) with a 582/15 band pass filter. Sorted cells were collected in media containing 20% FBS, 40% PBS, and 40% OPTI-MEM for differentiation and transplantation studies.

### Transplantation Studies

NIHIII mice were anesthetized with a ketamine/xylazine mixture (135 mg/kg ketamine and 15 mg/kg xylazine). A 3 mm mid-diaphyseal bone segment was removed from the femur using a bone saw. A plastic triangular shaped pin was inserted within the intramedullary space to provide internal support, while an external fixator was attached to the outer periosteal bone surface of the bone with resorbable sutures (Vicryl 6-0, Ethicon; polyglactin; Owens &Minor). The critical size defect was loaded with a Healos scaffold (DuPuy Spine) containing a basement membrane extract (Cultrex) - BMSC mixture. The scaffold contained 1×10^6^ stromal cells derived from *OEC*/+;*Ai9*/+ mice that were cultured for 5 days prior to transplantation. Finally, muscle and skin were sutured to their original positions. Bone repair was monitored over an 8 week period by X-ray (Faxitron Bioptics). After 8 weeks, tissue was harvested for histological imaging. Transplantation studies were biologically replicated two times with comparable outcomes.

### Microscopy and Imaging

Cells in culture and tissue sections were imaged using a Zeiss Observer Z.1 inverted microscope. For imaging Ai9 Cre reporter expression, multiple tissue sections (n = 5+/sample) from at least three mice were evaluated and representative data was imaged. Fluorescence was detected using the following filter sets (Chroma Technology): Tomato (ET545/30 Ex, ET620/60 Em,T570lp beam splitter), Alexa Fluor 488 and Dylight 488 (HQ500/20 Ex; HQ 535/30 Em, Q515lp beam splitter). Images were captured using an Axiocam MRc digital camera and Axiovision software (Zeiss).

## Results

### Osterix-EGFPCre Activation of Ai9 Broadly Marks Cells throughout the Bone Marrow Stroma

**Figure 1:**
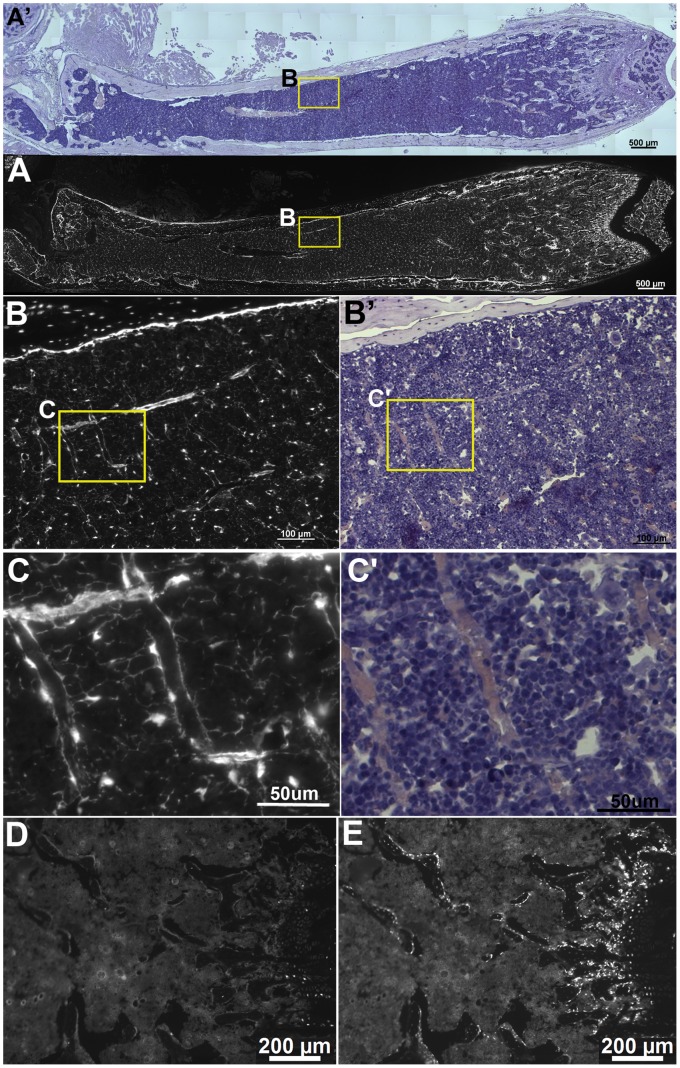
Visualization of *OEC* Mediated Cre Reporter Expression Inside the Bone Marrow. (**A–C**) In addition to activating Cre reporter expression (shown in white) in cells of the osteoblast lineage, *OEC* mice resulted in the broad marking of non-hematopoietic cells within the bone marrow compartment. (**A’–C’**) Hematoxylin counterstained tissue sections corresponding to images A–C. At progressively higher levels of magnification (**B** and **C**, yellow dashed box regions), Cre reporter expression is observed in cells that contribute to the stroma throughout the bone marrow. (**C, C’**) At high magnification many Cre reporter expressing cells retain long narrow processes reminiscent of bone marrow reticular cells and line vascular sinuses. (D) Expression of the Osterix EGFPCre reporter was not detected in trabecular bone osteoblasts or bone marrow in 6 week old femurs. However, expression of Osterix was detected by immunostaining in trabecular bone osteoblasts and osteocytes (E).

Femoral bone tissue sections were examined from 6 week old Osterix-EGFPCre (OEC)/Ai9 dual transgenic mice. The *Ai9* Cre reporter conditionally expresses the red fluorescent protein tdTomato in response to Cre recombinase activation. Cre reporter fluorescence in the hind limbs of adult mice was restricted to the bony elements and absent from soft tissues outside the bone cortices (Fig. 1, A-C, white fluorescence). However, inside the bone marrow, many cell types in addition to cells of the osteoblast lineage retained Cre reporter expression (Fig. 1A–C,A’–C’). Given this lineage tracing approach, the cell populations retaining Cre reporter expression were understandably heterogeneous. However, many bone marrow cells morphologically appeared to be stromal cells, i.e. they contained fine, dendritic-like processes and lined vascular sinusoids (Fig. 1B–B’,C–C’), similar to what has been previously reported [2]. FACS analysis indicated that 5–7% of the flushed bone marrow cell population retained Cre reporter expression (data not shown), indicating that a rather high percentage of the bone marrow cell population has been marked by *OEC* activity. Surprisingly, EGFP expression generated by the Osterix-EGFPCre transgene was largely undetectable within the endosteum and marrow compartment of the femur (Fig. 1D), despite our ability to detect endogenous Osterix expression in osteoblasts and osteocytes within trabecular bone by immunostaining (Fig. 1E).

### Prolonged Temporal Tracing of Osterix-CreERt Labeled Embryonic Precursors Reveals the Persistence of their Progeny inside the Bone Marrow

**Figure 2:**
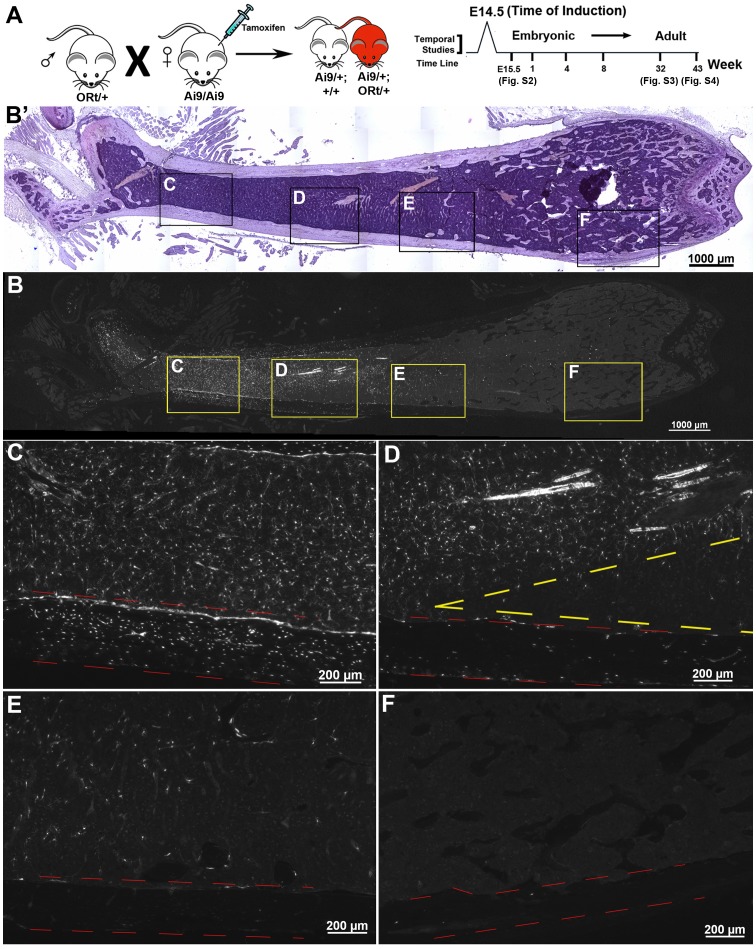
Temporal *Osterix* Fate Mapping of Embryonic Precursors Reveals their Persistence in the Bone Marrow. (**A**) *Osterix*-CreERt mice were intercrossed with *Ai9* mice for temporal fate mapping. A single tamoxifen injection was given to pregnant females at E14.5 of embryonic development. F1 offspring were harvested at E15.5 and at 1, 4, 8, 32, and 43 weeks of age. (**B**) Tissue section of a 8 week old femur showing Cre reporter expression (shown in white) favoring the proximal end of the bone. (**B’**) Corresponding hematoxylin counterstained femur to that shown in B. (**C–F**) Higher magnification images of bone marrow and adjacent cortical bone (traced by red dashed lines) taken along the proximal distal axis of the femur. At the proximal end (**C**) Cre reporter expressing cells were abundant within the bone marrow and cortical bone. Moving distally along the femur (**D**) a noticeable gap in Cre reporter expressing cells is observed in the cortical bone (red dashed lines) and adjacent marrow (yellow dashed lines), while the center of the marrow still retains an abundant amount of Cre reporter expressing cells. (**E, F**) At further distal locations along the femur substantially less, if any, Cre reporter expressing cells are detected.

The broad detection of *OEC* activated Cre reporter expressing cells within the bone marrow along with the absence of EGFPCre expression in 6 week old mice suggested that these cell populations were the progeny of earlier *Osterix* expressing progenitor cells that now potentially sustain themselves. To test this possibility, we obtained *Osterix*-CreERt (ORt) mice to carry out inducible fate mapping via tamoxifen treatment. Control experiments were first carried out to confirm the functionality of this animal model (Fig. S1). For embryonic fate mapping, pregnant females from *Osterix*-CreERt (*ORt*) and Ai9 intercrosses were given a single dose of tamoxifen at E14.5, when *Osterix* expression begins in the hind limb. To provide evidence for the selective nature of CreERt activation, we sectioned through hind limbs one day after induction (E15.5), which showed that *Ai9* Cre reporter expression was restricted to cells located in the mid-diaphyseal perichondrial region and developing marrow space (Fig. S2A–A’). No Cre reporter expression was observed in the growth plate. Thereafter, we evaluated the persistence of E14.5 tamoxifen induced cells in 1 week (Fig. S2B–D), 1 month (not shown), 2 month (Fig. 2), 7 month (Fig. S3) and 10 month (Fig. S4) old animals. At all ages observed, cells retaining Cre reporter expression persisted in the bone marrow indicating that the progeny of *Osterix* expressing embryonic precursors contributed to long-lived bone marrow cell populations.

### Prolonged Temporal Tracing Provides Insight into the Assembly of the Stroma during Bone Growth

Embryonic fate mapping also revealed the presence of Cre reporter expressing cells that are located largely at the proximal end of the femur (Fig. 2 B–B’,C–F). We believe the asymmetrical distribution of fate mapped progeny can be explained by the manner in which the femur grows after tamoxifen injection. As tamoxifen levels decrease, subsequent unmarked cells, likely from the perichondrium, continue to contribute to the formation of the bone marrow and bone tissue. For the femur, temporal tracing indicated greater longitudinal bone growth at the distal growth plate relative to the proximal growth plate. Additionally, the distal end of the femur gradually widens as it elongates, leaving Cre reporter expressing cells within the center of the marrow (Fig. 2D, yellow dashed line).

The ability to visualize the regional distribution of *Osterix-Cre* labeled progeny within the bone marrow (Figs. 2, S3, S4) is informative as to how the assembly of the stroma occurs. Embryonic precursors, presumably from the perichondrium, enter into the bone marrow at progressive stages of bone growth. The stage of development and the timing of perichondrial invasion into the bone marrow will largely determine the region of the bone marrow it will occupy.

### Osterix-Cre Fate Mapping Marks a Variety of Cell Types within the Bone Marrow Stroma

Constitutive (OEC) and temporal (Ort) tracing resulted in the labeling of a variety of cell lineages inside the bone marrow. To further characterize these cell types, we carried out immunostaining studies in bone tissue sections from 2 month old animals. Since *Osterix* is actively expressed in the cells of the osteogenic lineage, we focused on non-osseous cell types retaining Cre reporter expression within the bone marrow compartment.

**Figure 3:**
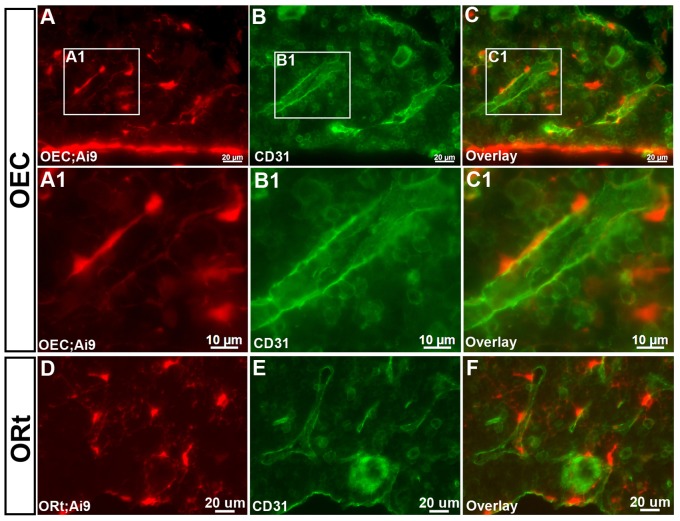
*Osterix*-Cre Fate Mapping Marks Bone Marrow Perivascular Stromal Cells. Immunostaining for the endothelial cell marker, CD31 revealed that *OEC* (**A–C1**) and *ORt* (**D–F**) fate mapping does not result in Cre reporter expression in endothelial cells, but in a perivascular stromal cell population associated with vascular sinuses of the bone marrow. (**A, A1,** and **D**) Cre reporter expression (shown in red). (**B, B1,** and **E**) CD31 immunostaining (shown in green). (**C, C1,** and **F**) Overlay showing association of Cre reporter expressing cells with bone marrow vasculature.

A sinusoidal endothelial network permeates the bone marrow and perivascular stromal cells associate with these tissues in order to support different hematopoietic events. To further define the identity of this cell population, immunostaining for CD31 was carried out, which identifies endothelial cells lining the vascular sinuses. *OEC/Ai9* (Fig. 3A–C,A1–C1) and *ORt/Ai9* (Fig. 3D–F) marked perivascular cells were distinct from endothelial cells, but were commonly located at the periphery of vascular sinusoids. *OEC* and *ORt* marked perivascular cells also immunostained positive for CD106/Vcam1 and, while CD106 has been referred to as a pan-reticular marker, in our hands it was by no means a selective marker for only this cell population (data not shown).

**Figure 4:**
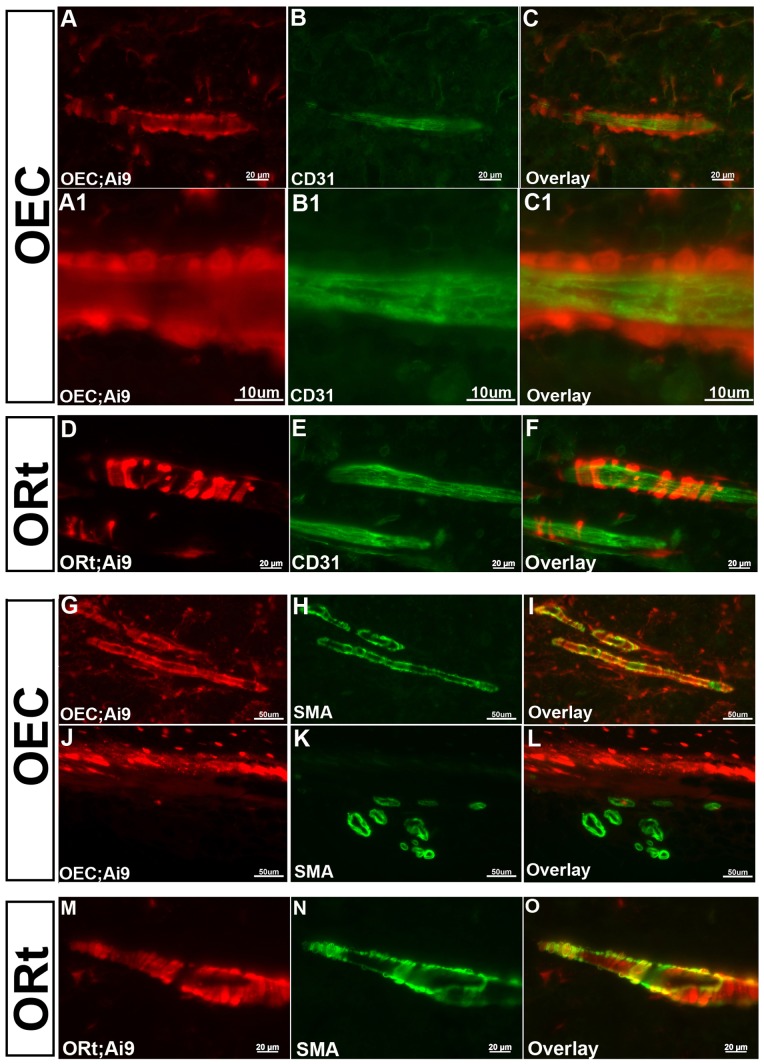
*Osterix*-Cre Fate Mapping Marks Bone Marrow Vascular Smooth Muscle. Cre reporter expression activated by *OEC* (**A–C1** and **G–L**) and *ORt* (**D–F** and **M–O**) mice was expressed in vascular smooth muscle cells, which encompassed arterioles running longitudinally through the center of the bone marrow. Vascular smooth muscle was identified through a combination of CD31 (**A–F**) and smooth muscle actin alpha 2 (SMA) (**G–O**) immunostaining. The marking of vascular smooth muscle was restricted to the bone marrow compartment as vascular smooth muscle cells lying just outside the cortical bone surface did not contain any Cre reporter expression (**J–L**). (**A, A1, D, G, J, M**) *Ai9* Cre reporter expression shown in red. (**B, B1, E**) CD31 immunostaining shown in green. (**H, K, N**) SMA immunostaining shown in green. (**C, C1, F, I, L, O**) Merged images.

A second bone marrow vascular cell population retaining *OEC/Ai9* (Fig. 4A–C,A1–C1,G–L) and *ORt/Ai9* (Fig. 4D–F,M–O) generated Cre reporter expression was smooth muscle cells, which encompassed the arterioles present within the bone marrow. Endothelial tubes, which stained for CD31 did not show Cre reporter expression (Fig. 4A–C,A1–C1,D–F), but smooth muscle surrounding the blood vessels, as identified by α-Smooth Muscle Actin 2 immunostaining (Fig. 4G–O), did retain Cre reporter expression. Interestingly, smooth muscle lined blood vessels in close proximity to the outer cortical bone surface (Fig. 4J–L) did not have *OEC/Ai9* Cre reporter expression, suggesting a distinct origin to smooth muscle cells within the bone marrow relative to those just outside the cortical bone.

**Figure 5:**
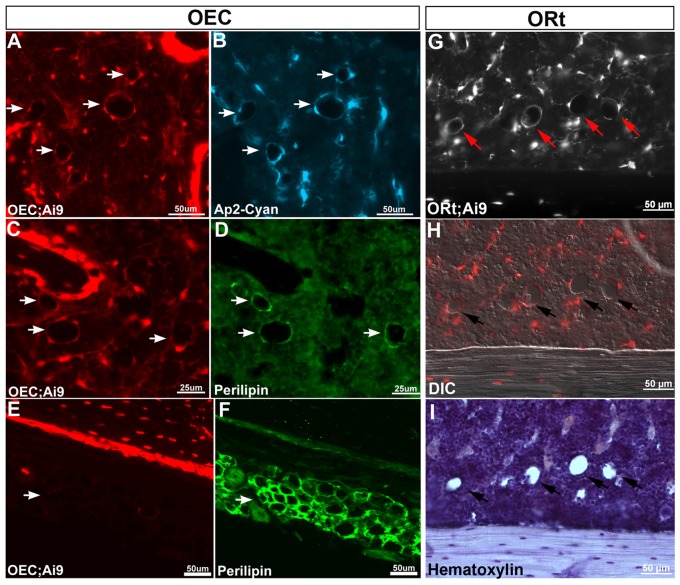
*Osterix*-Cre Fate Mapping Marks Bone Marrow Adipocytes. *OEC* (**A–F**) and *ORt* (**G–I**) activated *Ai9* Cre reporter expression was observed in bone marrow adipocytes, which appear as empty, round-shaped cells in tissue section. Cre reporter expressing bone marrow adipocytes (**A, C, E, G, H**) were identified using *aP2-EGFPcyan* reporter mice (**B**, blue fluorescent adipocytes noted by white arrows), immunostaining for Perilipin (**D**, green fluorescent adipocytes noted by white arrows), and hematoxylin counterstained tissue sections revealed round empty holes (**I,** black arrows). The marking of adipocytes was restricted to the bone marrow compartment as adipose tissue located just outside the bone cortices retained no Cre reporter expression (**E, F**).

*OEC/Ai9* (Fig. 5A–F) and *ORt/Ai9* (Fig. 5G–I) Cre reporter expression could also be detected in bone marrow adipocytes. Adipocytes in the bone marrow have a rounded cell morphology that after histological processing appeared as round empty holes. Adipocytes were positively identified by intercrossing OEC/*Ai9* mice with *Fatty Acid Binding Protein 4 (aP2-GFPcyan)* reporter mice (Fig. 5A,B, white arrows) and by immunostaining for Perilipin (Fig. 5C,D, white arrows). Temporal *ORt/Ai9* tracing also resulted in the marking of a subpopulation of bone marrow adipocytes seen in the distal tibia (Fig. 5G–I, red and black arrows). However, adipose tissue in close proximity to the outer cortical bone retained no *OEC/Ai9* Cre reporter expression (Fig. 5E,F), again suggesting distinct origins for adipocytes present inside the bone marrow relative to those outside of bone tissue.

**Figure 6:**
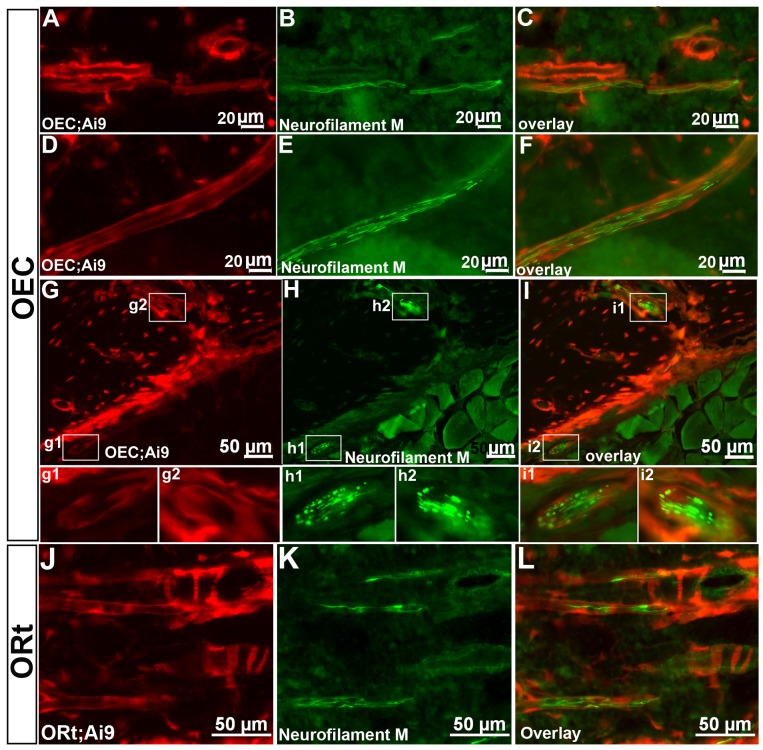
*Osterix*-Cre Fate Mapping Marks Bone Marrow Perineural Cells. *OEC* (**A-i2**) and *ORt* (**J–L**) activated *Ai9* Cre reporter expression (red) was observed in perineural cells that formed tubes around nerve fibers (green) located in the bone marrow compartment (A–F and J–L), channels of the cortical bone (g2, h2, i2) and adjacent to the outer periosteum (g1, h1, i1). Nerve fibers were identified by immunostaining for Neurofilament M (B, E, H, h1, h2, K, green).

Sympathetic and sensory nerve fibers innervate the bone marrow. Therefore, we examined *OEC* (Fig. 6A–I,g1-i2) and *ORt* (Fig. 6J–L) generated Cre reporter expressing cells relative to Neurofilament M immunostaining. Interestingly, *Osterix* generated Cre reporter expression was observed in perineural tubes surrounding neurofilament M+ nerve fibers within the bone marrow (Fig. 6A–C,D–F,J–L). The perineurium is a layer of connective tissue that encloses myelinated nerve fibers. We also detected nerve fibers entering the cortical bone through channels (Fig. 6G–I) and in proximity to the outer cortical bone surface that were enclosed by Cre reporter positive tubes as well (Fig. 6g1,g2,h1,h2,i1,i2). However, nerve fibers running through muscle tissue adjacent to the cortical bone did not have Cre reporter positive perineurial cells (data not shown).

### Bone Marrow Cells Derived from Osterix-EGFPCre/Ai9 Cre Reporter Mice Mark an Adherent Stromal Cell Population

**Figure 7:**
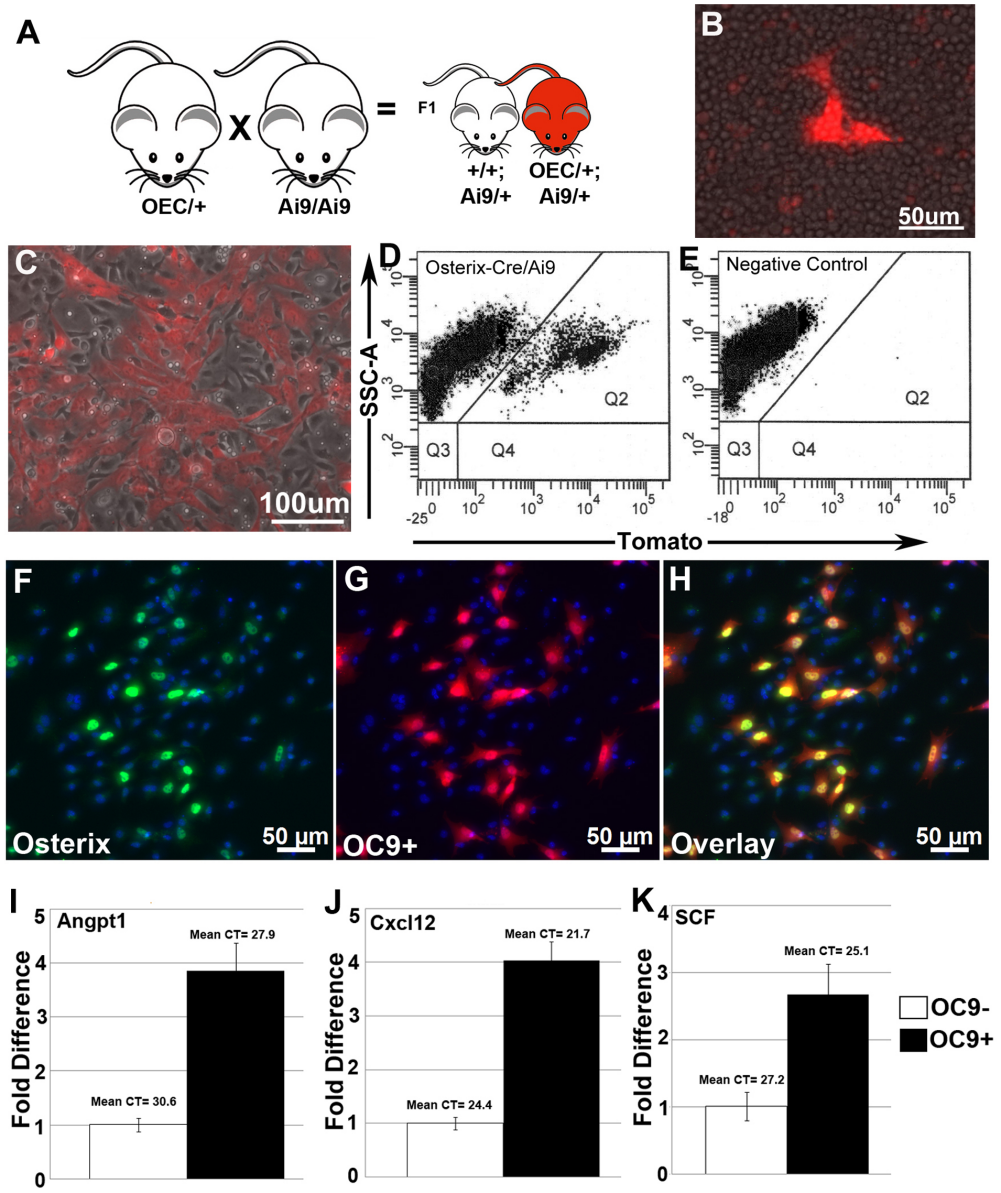
Constitutive *Osterix*-Cre Fate Mapping Marks a Bone Marrow Stromal Cell Population. (**A**) The intercross of *Osterix*-Cre mice with *Ai9* Cre reporter mice generates dual transgenic offspring containing *Osterix*-Cre and the *Ai9* Cre reporter (red mouse). (**B**) Cre reporter expressing cells were present in the bone marrow flush, a subset of which started to adhere to the tissue culture plate within the first 24 hours. (**C**) By day 5 of stromal culture OC9+ cells (the *Osterix*-Cre mediated Cre reporter expressing population) represents a subpopulation of the adherent cell fraction and retains a mesenchymal cell morphology. (**D, E**) FACS analysis from day 5 stromal cultures showed that OC9+ cells represent 15–20% of the total cell population. (**F–H**) Immunostaining for Osterix in day 5 bone marrow stromal cultures resulted in nuclear localized staining (green) that was restricted to ∼68% of OC9+ cells (red). (**I–K**) OC9 positive and negative cells fractions were harvested by FACS after 5 days in culture. Quantitative RT-PCR revealed modest up-regulation of known perivascular stromal gene markers Angpt1, Cxcl12, and SCF in OC9+ cells.

The persistence of ORt/Ai9 marked bone marrow cell types and their tracing into multiple cell lineages, suggested that Osterix expressing embryonic precursors may give rise to adult progenitor cell types within the bone marrow that are responsible for maintaining different cellular components of the stroma. To provide more evidence for the Osterix-Cre marking of bone marrow progenitors, stromal cultures were prepared from OEC/Ai9 dual transgenic offspring (Fig. 7A). When total bone marrow was flushed from *OEC/Ai9* mice and plated at non-clonal densities, fluorescently marked cells expressing the *Ai9* tdTomato reporter were present and, within the first 24 hours, a subpopulation of *OEC/Ai9* (OC9+) cells started to attach to tissue culture plastic (Fig. 7B) and increased thereafter. OC9+ cells retained a fibroblast-like cell morphology and expanded in concentric colonies (Fig. 7C). FACS analysis of the adherent cell fraction from day 5 stromal cultures showed that ∼20% of the total cell population was OC9+ (Fig. 7D,E). Additionally, despite our inability to visually detect EGFP fluorescence, endogenous Osterix was detected at low levels by immunostaining at days 3 and 5 of culture in ∼68% of the OC9+ adherent cell fraction (Fig. 7F–H). Additionally, we also investigated the expression of the perivascular stromal markers *Angiopoietin 1* (*Angpt1*), *Chemokine CXC Ligand 12* (*Cxcl12*), and *Stem Cell Factor* (*SCF*), which have been shown by others to be expressed in multipotent stromal cells [13], [17], [18]. Quantitative PCR revealed that activities of all 3 of these genes were modestly up-regulated in the OC9+ cell fraction relative to the negative cell fraction (Fig. 7I–J).

### The OC9+ Cell Population Displays Mesenchymal Multipotency In Vitro

**Figure 8:**
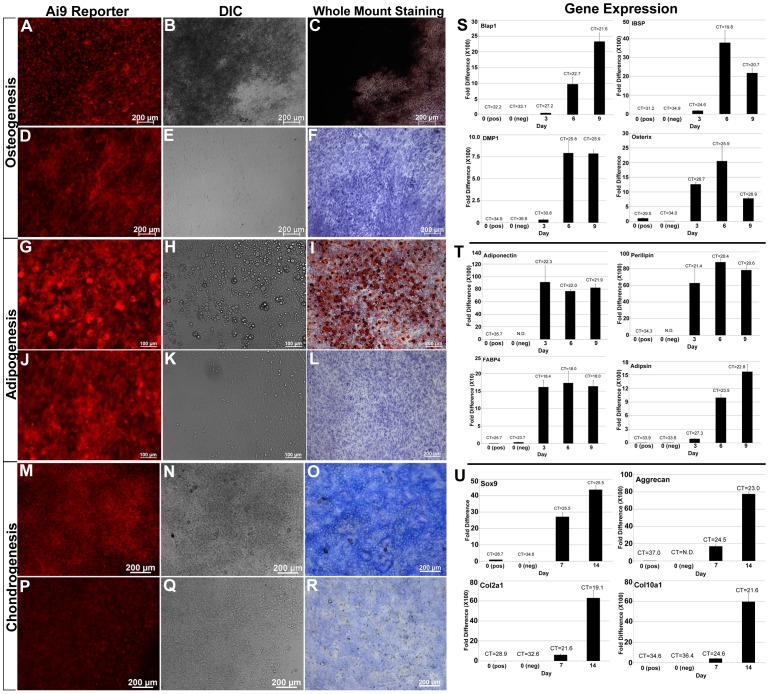
In Vitro Differentiation of OC9+ Cells Demonstrates Their Multipotent Properties. OC9+ cells were FACS isolated from day 5 stromal cultures, replated in culture, and differentiated into osteoblasts (**A–F, S**), adipocytes (**G–L, T**), and chondrocytes (**M–R, U**). (**A, D, G, J, M, P**) Detection of *Ai9* Cre reporter expression. (**B, E, H, K, N, Q**) Imaging of cultures under DIC optics. (**C, F, I, L, O, R**) Imaging of whole mount staining of cultures. (**S–U**) Temporal assessment of differentiation by quantitative RT-PCR. (**A–C**) OC9+ cells differentiated under osteogenic conditions produced a robust mineralized matrix that could be visualized under DIC optics (**B**) and by von Kossa staining (**C**). (**D–F**) OC9+ cells grown in the absence of osteogenic conditions did not produce a mineralized matrix and did not stain by von Kossa. (**S**) Osteoblast differentiation was examined at days 0, 3, 6, and 9 of culture. All osteogenic gene markers were dramatically up-regulated during differentiation including *Osteocalcin* (*Bglap1*), *Bone Sialoprotein* (*IBSP*), *Dentin Matrix Protein 1* (*DMP1*), and *Osterix*. (**G–I**) OC9+ cells differentiated under adipogenic conditions generated cells containing large lipid vesicles that could be easily observed under DIC optics (**H**) and were stained by Oil Red-O (**I**). (**J–L**) Control cultures grown under non-adipogenic conditions did not display large lipid vesicles (**K**) or stain with Oil Red-O (**L**). (**T**) Adipogenic differentiation was examined at days 0, 3, 6, and 9 of culture. All adipogenic gene markers were dramatically up-regulated during differentiation including *Adiponectin*, *Perilipin*, *Fatty Acid Binding Protein 4* (*FABP4*), and *Adipsin*. (**M–O**) OC9+ cells differentiated under chondrogenic conditions generated areas of condensing chondrocytes that were noticeable under DIC optics (**N**) and stained strongly positive for Alcian blue (**O**). (**P–R**) Control cultures grown under non-chondrogenic conditions did not generate areas of condensing chondrocytes (**Q**) and did not stain for Alcian blue (**R**). (U) Chrondrogenic differentiation was examined at days 0, 7, and 14 of culture. All chondrogenic gene markers were dramatically up-regulated during differentiation including *Sox9*, *Aggrecan*, *Collagen type 2 alpha I* (*Col2a1*), and *Collagen type 10 alpha 1* (*Col10a1*).

To test the multipotency of adherent OC9+ cells, we carried out *in vitro* differentiation studies down the osteoblast (Fig. 8A–F,S), adipocyte (Fig. 8G–L,T), and chondrocyte (Fig. 8M–R,U) cell lineages. Prior to differentiation, OC9+ cells were FACS isolated from day 5 stromal cultures and plated as confluent spots as previously described [19], [20].

For osteoblast differentiation, matrix deposition and von Kossa staining could be observed in OC9+ cells cultured in the presence of ascorbic acid and β-glycerol phosphate (Fig. 8B,C). No matrix or mineral deposition was detected when OC9+ cells were grown under non-osteogenic conditions (Fig. 8E,F). Quantitative RT-PCR was carried out on day 5 FACS isolated OC9+ (0 pos), OC9− (0 neg) cells and cells from days 3, 6, and 9 of osteoblast differentiation (Fig. 8S). *Osteocalcin* (*Bglap1*), *Bone Sialoprotein* (*IBSP*), *Dentin Matrix Protein 1* (*DMP1*), and *Osterix (Sp7)* gene expression were all substantially up-regulated during differentiation (fold difference is X100 for *Bglap1*, *IBSP*, and *DMP1*).

When OC9+ cells were differentiated under adipogenic conditions, large lipid vesicles could be observed under DIC optics (Fig. 8H), which could also be detected by Oil Red O staining (Fig. 8I). OC9+ cells grown in the absence of adipogenic inducers showed no large lipid vesicles or Oil Red O staining (Fig. 8K,L). Quantitative RT-PCR was carried out on day 5 FACS isolated OC9+ (0 pos), OC9− (0 neg) cells, and cells from days 3, 6, and 9 of adipogenic differentiation (Fig. 8T). The adipocyte gene markers *Adiponectin (Adipoq)*, *Perilipin (Plin1)*, *Fatty Acid Binding Protein 4* (*Fabp4*), and *Adipsin (Cfd)* were all substantially up-regulated during differentiation (fold difference is X100 for *Adiponectin, Perilipin*, and *Adipsin* and X10 for *FABP4*).

For chondrocyte differentiation, regional condensations formed during the 14 day differentiation period and cultures stained positive for alcian blue (Fig. 8N,O). Under control conditions, faint background alcian blue staining was observed (Fig. 8R). Quantitative RT-PCR was carried out on day 5 FACS isolated OC9+ (0 pos), OC9− (0 neg) cells, and day 7 and 14 OC9+ differentiated cells (Fig. 8U). *Sox9*, *Aggrecan (Acan)*, *Collagen type 2 alpha 1* (*Col2a1*), and *Collagen type 10 alpha 1* (*Col10a1*) were all substantially up-regulated upon differentiation (fold difference X100 for *Aggrecan*, *Col2a1*, and *Col10a1*). Collectively, these in vitro differentiation studies provided evidence that the adherent OC9+ bone marrow derived cell population, displayed BMSC-like multipotency.

### Transplantation of OC9+ Cells Revealed Their Ability to Differentiate into Many of the Same Cell Types Identified by Fate Mapping

**Figure 9:**
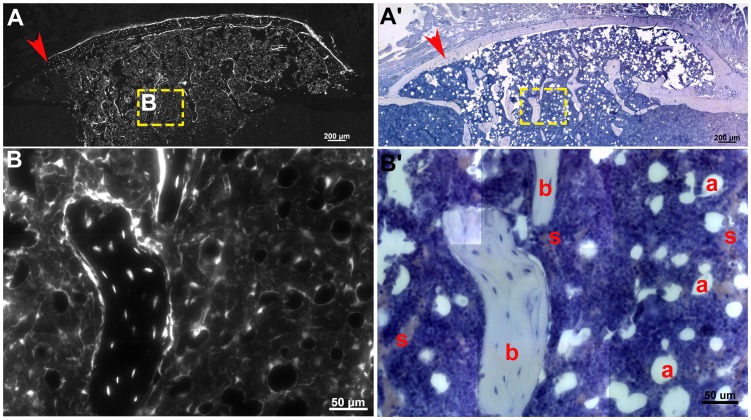
Transplantation of OC9+ Cells Demonstrates Their Multipotent Properties. Stromal cells derived from *Osterix-Cre*/*Ai9* mice were cultured for 5 days, loaded into a scaffold, and transplanted into a femoral skeletal defect. (**A**) Skeletal healing of the defect was monitored by x-ray over a 6 week period. (**B, D**) Detection of OC9+ cellular progeny (red) in a femoral tissue section 6 weeks after transplantation. (**C, E**) The same tissue section shown in **A** and **D** now counterstained with hematoxylin. (**B**) A low magnification image showing OC9 cellular progeny (red) contributing to cortical and trabecular bone tissue detected by DIC imaging and hematoxylin counterstaining (purple). The yellow arrow denotes the boundary between host bone and donor bone. (**D, E**) In addition to differentiating into osteoblasts and making bone tissue, higher magnification reveals that OC9+ cells contribute to bone marrow adipocytes within the marrow and perivascular cells lining the vascular sinusoids (B = bone, F = adipocytes, and S = vascular sinusoid).

To test for the multipotency of adherent OC9+ cells in vivo, day 5 bone marrow stromal cultures derived from *OEC/Ai9* mice were FACS isolated and transplanted into a femoral defect (Fig. 9). In this skeletal defect model, a 3 mm piece of bone tissue was removed from the center of the femur and a scaffold preloaded with OC9+ cells was transplanted between the separated proximal and distal femur bones. Eight weeks after transplantation the femur was harvested for histological examination (Fig. 9). Since *OEC/Ai9* labeled stromal cells retain ubiquitous Cre reporter expression, the contribution of OC9+ cells could be traced after transplantation. At low magnification, a broad region of cells containing Cre reporter expression was observed in the center of the femur (Fig. 9A,A’). OC9+ cells formed an outer cortical bone that integrated with host tissue (red arrow) and trabecular bone could be seen in the marrow space (Fig. 9A,B). At high magnification, a section through bone trabeculae shows osteoblasts and osteocytes retaining Cre reporter expression (Fig. 9B,B’). Additionally, *OC9+* stromal cells contributed to adipocytes within the bone marrow and reticular-like cells lining sinusoidal structures within the repair (Fig. 9B,B’, b = Bone, a = Adipocyte, and s = Sinusoid).

## Discussion

The transition from embryonic organogenesis to adult tissue homeostasis remains a complicated and poorly understood area of biology. However, an essential component of this process requires embryonic precursors to give rise to adult progenitor cells, which then contribute to the maintenance, turnover and repair of tissue. During development of the endochondral skeleton, we showed that embryonic precursors marked by *Osterix* fate mapping populate the bone marrow and give rise to long-lived progeny that trace into a variety of cell lineages that contribute to the makeup of the bone marrow stroma. Moreover, our studies provide evidence that *Osterix* fate mapping resulted in the marking of adult progenitor cells within the bone marrow that retain the potential to differentiate into many of the same cell lineages traced by fate mapping. Below, we discuss the implications of these results with regard to understanding bone growth, the marrow environment, and bone marrow progenitor cells.

### The Perichondrium: The Likely Source of Osterix Cre Labeled Bone Marrow Cells

Past studies have provided evidence that during endochondral bone formation cellular precursors derived from the perichondrium migrate into the bone marrow and contribute to the formation of the bone marrow stroma [1], [2]. However, lineage analysis has also shown that cells derived from the cartilage growth plate also contribute to cells within the marrow compartment [22]. Since *Osterix* is expressed in the mid-diaphyseal region of the perichondrium and in maturing chondrocytes of the growth plate, the origin of *Osterix*-Cre marked bone marrow cells may be debated. However, previous work has scrutinized the perichondrial versus growth plate question of origin and has provided more compelling evidence that the perichondrium is a major contributor to the bone marrow (Maes et al., 2010). Additionally, past studies from our lab have examined the entry of hypertrophic chondrocytes into the trabecular bone marrow region by visualizing Col10a1-Cherry reporter expression in conjunction with osteoblast reporter mouse models [23]. These latter studies revealed the chaotic nature of embryonic growth plate degradation as it transitions into trabecular bone, but did not support the belief that hypertrophic chondrocytes were a major source for different cellular components of the stroma. Our assessment of ORt activated Ai9 Cre reporter expression at E15.5, one day after tamoxifen induction, revealed Cre reporter expression in cells located in the perichondrium and bone marrow, with little Cre reporter expression detected in the growth plate (Fig. S2). Therefore, consistent with past work [1], [2], we believe that the bone marrow cells retaining Cre reporter expression originated from the perichondrium.

### Bone Growth and Formation of the Stroma are Coordinated Processes

It is well established that the growth of endochondral bones is a coordinated process between chondrocyte and osteoblast differentiation. Our studies suggest that proper development of the stroma is also coordinated with bone growth. First, a common source of embryonic precursors labeled by Osterix-Cre activity contributed to a variety of cell lineages within the stroma in addition to cells of the osteoblast lineage. Second, temporal tracing of embryonic precursors showed how the timing of perichondrial invasion into the bone marrow will largely determine the region of the bone marrow it will occupy. A reason for the coordinated development between bone and its stroma likely has to do with the formation of cortical bone. As this outer shell forms during bone elongation a physical barrier is created that highly limits the manner in which perichondrial cells can find entry into the bone marrow. Thus, perichondrial precursors must migrate into the bone marrow prior to cortical bone formation.

### The Stroma: An Organized Structure

Because the bone marrow largely consists of hematopoietic cells, it is easy to conceive of the bone marrow as being a fluid compartment. However, one of the surprising outcomes of this study related to the limited movement of temporally traced perichondrial derived cells within the bone marrow compartment. Ten months after tamoxifen induction, historically marked cells remained near the proximal end of the femur and did not spread into other areas of the bone marrow. Thus, while cells of the hematopoietic lineage actively move through blood vessels and sinusoids of the bone marrow, many cell types of the stroma appear to have limited motility. One rational explanation for why cellular components of the stroma may have limited movement is highlighted by recent studies, which used a variety of Cre lines to molecularly dissect different hematopoietic niches inside the bone marrow [24], [25], [26]. These studies reveal how niches located in different regions of the bone marrow have distinct roles in hematopoiesis. Thus, it is likely that most cell types contributing to the structure of the stroma need to keep their position in order to maintain distinct niches within the bone marrow.

### Fate Mapping Reveals the Uncommitted Nature of Osterix Expressing Progenitor Cells

A fundamental aspect of cellular differentiation relates to understanding levels of multipotency versus commitment within a developmental lineage. Past studies have unequivocally demonstrated the functional importance of *Osterix* in osteoblast formation [27]. In this regard, the onset of *Osterix* expression has always been associated with osteoblast commitment. Contrary to these functional studies, the genetic tools used to trace *Osterix* expressing cells provide evidence for a much more complex regulation of cellular differentiation. Our studies showed that embryonic precursors marked by temporal *ORt* fate mapping were not restricted to the osteoblast lineage and contributed to multiple cell types within the bone marrow stroma (Figs. 3, 4, 5, 6). Furthermore, OEC fate mapping resulted in the labeling of adult stromal cells, many of which expressed Osterix, but as a population retained the capacity to differentiate into a variety of cell lineages. Consistent with this work, recently it was shown that when Cre recombinase activity was induced in adult ORt mice, some bone marrow adipocytes retained Cre reporter expression in addition to cells of the osteoblast lineage [28]. This suggests that some of the cells retaining Osterix expression even at adult ages are early bone marrow progenitors with multi-lineage potential. Collectively, these findings considerably expand the repertoire of cell types that *Osterix* expressing progenitor cells may become while inside the bone marrow at both embryonic and adult ages. Recently, we reported on the generation of an Osterix-Cherry reporter mouse model that showed low reporter expression in a population of bone marrow cells in proximity to bone surfaces [29]. We speculate that low Osterix expression identifies an early bone marrow progenitor cell population that remains uncommitted to the osteoblast lineage.

### A Common Origin for Different Adult Bone Marrow Progenitor Cells?

Our studies suggest that Osterix-Cre fate mapping resulted in the labeling of a variety of progenitor cell types that are required to maintain different structures inside the stroma. The demonstration that transplanted OEC labeled stromal cells can differentiate into many of the same cell types observed by fate mapping further reinforces the belief that Osterix-Cre fate mapping does mark potentially diverse populations of adult bone marrow progenitors. In many ways, the OC9+ cell population retained the hallmark characteristics of a mesenchymal stem cell population. At the same time, we don’t know whether the OC9+ cell population is a compilation of different adult progenitors already predisposed to differentiate down a certain lineage or a cell population containing individual multipotent adult stem cells, or both. In support of these studies, recently OEC Cre mice were used to conditionally disrupt Cxcl12 in CAR (Cxcl12 Abundant Reticular) cells [26], which are broadly distributed throughout the marrow and have been shown to display adipo-osteogenic potential [13].

While the field of mesenchymal stem cell biology has sought to discover a unique marker gene to selectively identify multipotent bone marrow progenitors, a possible alternative to consider is that progenitor cells adopt different gene expression profiles based on their local environment, but retain some level of skeletal memory based on their common origin. Future studies will seek to further resolve and understand the bone marrow progenitor cell types marked by Osterix-Cre fate mapping.

## Supporting Information

Figure S1 Control Experiments Assessing *ORt* Function. **(A–K)** One month old mice were initially used to evaluate ORt functionality. *Ai9* Cre reporter expression was detected in femurs derived from *ORt/Ai9* mice injected with **(A)** 0.05 mg tamoxifen/gram weight and **(B)** 0.025 mg tamoxifen/gram weight. **(C)** No Cre reporter expression was detected in *ORt/Ai9* mice injected with an equivalent volume of the vehicle (corn oil). Additionally, no Cre reporter expression was detected in *ORt* **(D)** or *Ai9* **(E)** mice with tamoxifen injection. **(F–I)** Soft tissues derived from *ORT/Ai9* mice injected with 0.05 mg tamoxifen/gram weight (from same mouse as femur shown in A) (F. heart and lung tissue, G. spleen, H. liver, I. kidney). **(J, J’)** Image of femur tissue section shown in A. **(J)** Detection of Cre reporter expression, which appeared largely restricted to the osteoblast lineage. **(J’)** Corresponding hematoxylin counterstained tissue section to that shown in J. **(K, K’)** Image of femur tissue section shown in C. **(K)** No Cre reporter expression was detected in tissue sections of vehicle injected mice at one month of age. **(K’)** Corresponding hematoxylin counterstained tissue section to that shown in K. Note: All tissues were harvested 48 hours after injection. (L,L’,M,M’) While no leakage was observed in young mice, osteocyte selective leakage of CreERt activity was noticed in much older mice 39 weeks of age. Importantly, the bone marrow area retains no Cre reporter expressing cells. **(L’** and **M’)** Corresponding hematoxylin counterstained images of the same regions shown in L and M.
(TIF)

Figure S2 Examination of Cre Reporter Expression in E15.5 and 1 Week Old Bones after Tamoxifen Treatment at E14.5 of Embryogenesis. *ORt* activated *Ai9* Cre reporter expression (shown in white) following tamoxifen induction at E14.5 was examined at E15.5 **(A, A’)** and 1 week of age **(B–D)** in bone tissue sections. **(A, A’)** Tissue sections through an E15.5 femur showing Cre reporter expression (A, white) in cells along the outer perichondrium and within the newly forming marrow compartment. **(A’)** Corresponding hematoxylin counterstained tissue to that shown in A. **(B–D)** In 1 week old tibia sections (proximal end –top, distal end –bottom) the distribution of Cre reporter expressing cells appears with higher frequency at the distal end **(D, D’)** relative to the proximal end **(C, C’)** of the tibia.
(TIF)

Figure S3 Examination of Cre Reporter Expression in a 32 Week Old Femur after Tamoxifen Treatment at E14.5 of Embryogenesis. Cre reporter expressing cells persisted in the bone marrow of 32 week old mice and remain localized toward the proximal end of the femur. **(A)** Image of Cre reporter expression (white) and **(A’)** corresponding hematoxylin counterstained tissue section. (B-E) Regions of interest along the proximal-distal axis of the femur showing the reduction in Cre reporter expressing cells. (F, F’, G, G’) Many of the cells that persist in the bone marrow retain a reticular cell morphology.
(TIF)

Figure S4 Examination of Cre Reporter Expression in a 43 Week Old Femur after Tamoxifen Treatment at E14.5 of Embryogenesis. Cre reporter expressing cells persisted in the bone marrow of 43 week old mice and remain localized toward the proximal end of the femur. **(A)** Image of femur (distal end – top, proximal end - bottom) showing Cre reporter expressing cells (white). **(B–E)** Regions of interest along the proximal-distal axis of the femur showing the increase in Cre reporter expressing cells as one moves toward the proximal end of the bone.
(TIF)

Table S1 Oligonucleotides used in this study.
(DOC)
