# Supplementary figures and images for: Osterix-Cre Labeled Progenitor Cells Contribute to the Formation and Maintenance of the Bone Marrow Stroma
Source: PLoS One. 2013 Aug 8;8(8):e71318. doi: 10.1371/journal.pone.0071318 (PMC3738599; doi:10.1371/journal.pone.0071318)

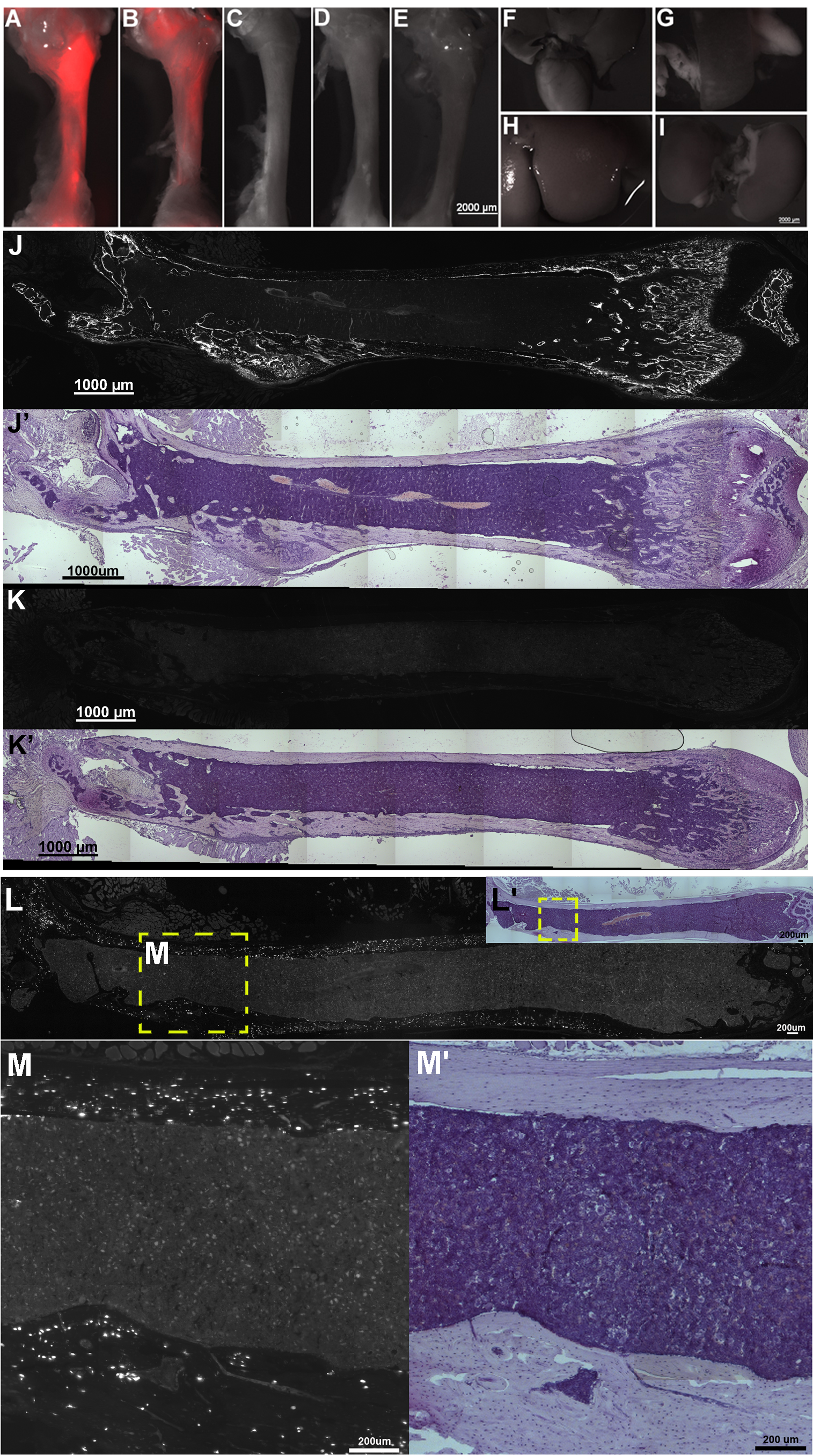

Supplement: Figure S1 — Control Experiments Assessing ORt Function. (A–K) One month old mice were initially used to evaluate ORt functionality. Ai9 Cre reporter expression was detected in femurs derived from ORt/Ai9 mice injected with (A) 0.05 mg tamoxifen/gram weight and (B) 0.025 mg tamoxifen/gram weight. (C) No Cre reporter expression was detected in ORt/Ai9 mice injected with an equivalent volume of the vehicle (corn oil). Additionally, no Cre reporter expression was detected in ORt (D) or Ai9 (E) mice with tamoxifen injection. (F–I) Soft tissues derived from ORT/Ai9 mice injected with 0.05 mg tamoxifen/gram weight (from same mouse as femur shown in A) (F. heart and lung tissue, G. spleen, H. liver, I. kidney). (J, J’) Image of femur tissue section shown in A. (J) Detection of Cre reporter expression, which appeared largely restricted to the osteoblast lineage. (J’) Corresponding hematoxylin counterstained tissue section to that shown in J. (K, K’) Image of femur tissue section shown in C. (K) No Cre reporter expression was detected in tissue sections of vehicle injected mice at one month of age. (K’) Corresponding hematoxylin counterstained tissue section to that shown in K. Note: All tissues were harvested 48 hours after injection. (L,L’,M,M’) While no leakage was observed in young mice, osteocyte selective leakage of CreERt activity was noticed in much older mice 39 weeks of age. Importantly, the bone marrow area retains no Cre reporter expressing cells. (L’ and M’) Corresponding hematoxylin counterstained images of the same regions shown in L and M. (TIF) [file pone.0071318.s001.tif]

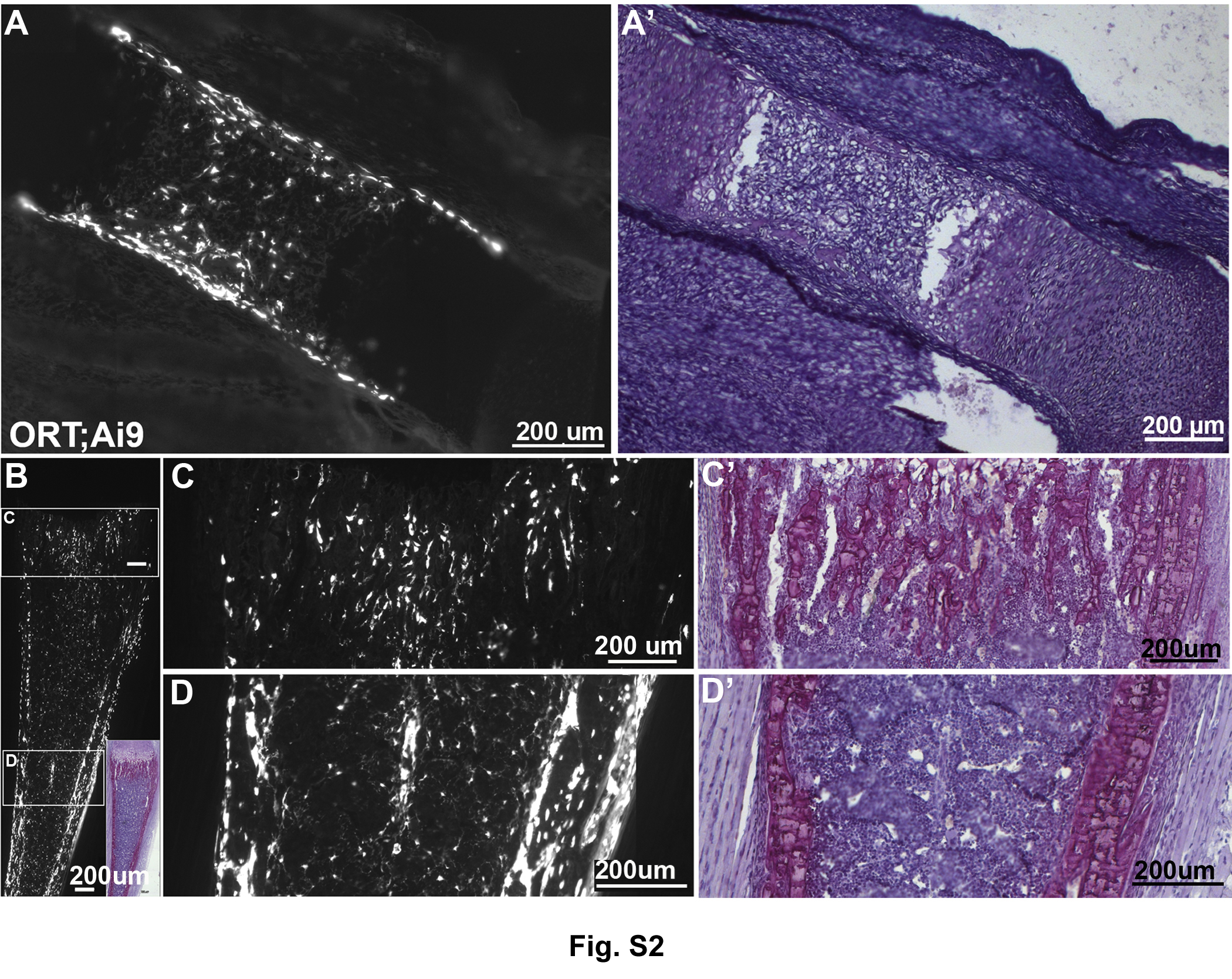

Supplement: Figure S2 — Examination of Cre Reporter Expression in E15.5 and 1 Week Old Bones after Tamoxifen Treatment at E14.5 of Embryogenesis. ORt activated Ai9 Cre reporter expression (shown in white) following tamoxifen induction at E14.5 was examined at E15.5 (A, A’) and 1 week of age (B–D) in bone tissue sections. (A, A’) Tissue sections through an E15.5 femur showing Cre reporter expression (A, white) in cells along the outer perichondrium and within the newly forming marrow compartment. (A’) Corresponding hematoxylin counterstained tissue to that shown in A. (B–D) In 1 week old tibia sections (proximal end –top, distal end –bottom) the distribution of Cre reporter expressing cells appears with higher frequency at the distal end (D, D’) relative to the proximal end (C, C’) of the tibia. (TIF) [file pone.0071318.s002.tif]

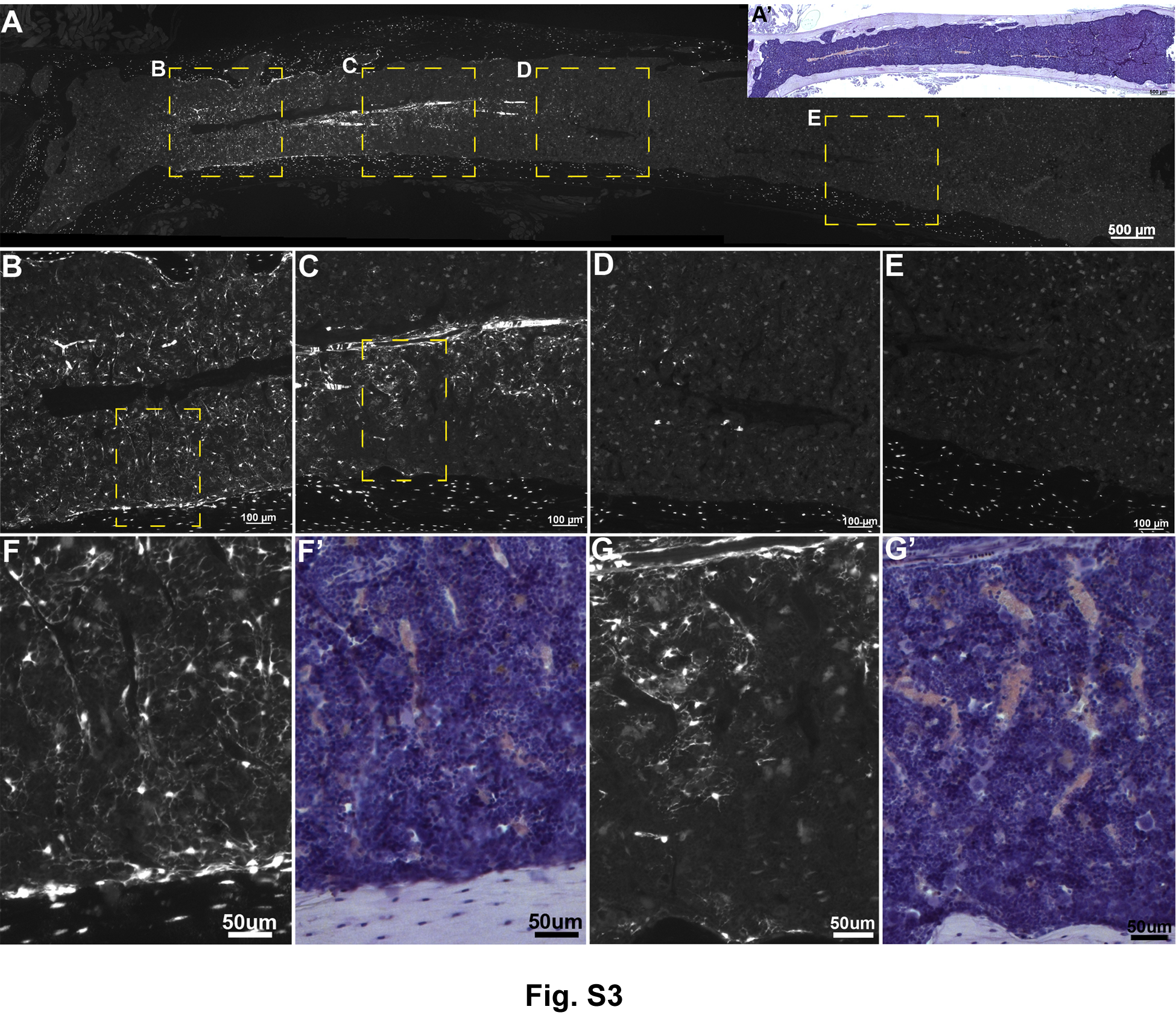

Supplement: Figure S3 — Examination of Cre Reporter Expression in a 32 Week Old Femur after Tamoxifen Treatment at E14.5 of Embryogenesis. Cre reporter expressing cells persisted in the bone marrow of 32 week old mice and remain localized toward the proximal end of the femur. (A) Image of Cre reporter expression (white) and (A’) corresponding hematoxylin counterstained tissue section. (B-E) Regions of interest along the proximal-distal axis of the femur showing the reduction in Cre reporter expressing cells. (F, F’, G, G’) Many of the cells that persist in the bone marrow retain a reticular cell morphology. (TIF) [file pone.0071318.s003.tif]

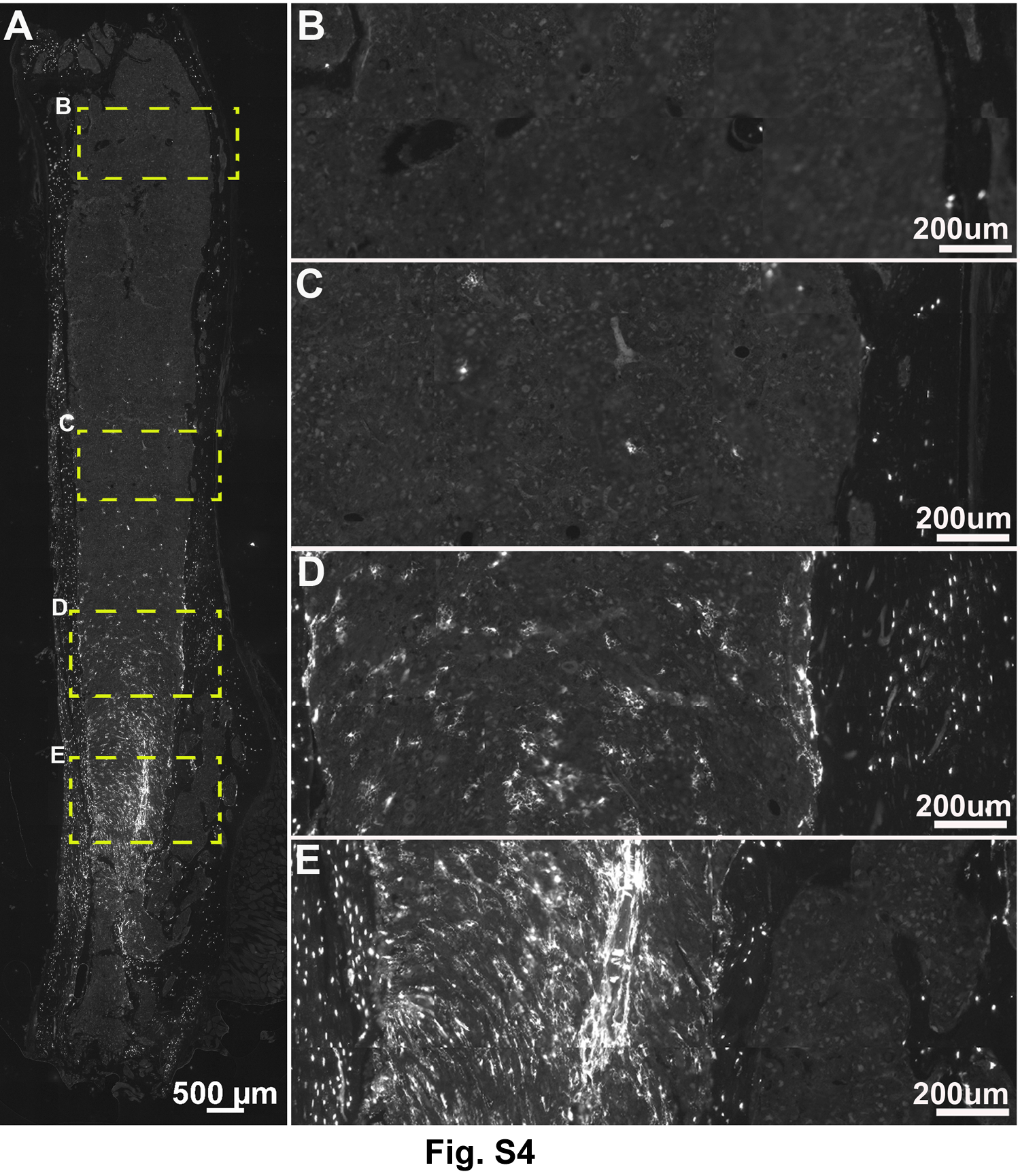

Supplement: Figure S4 — Examination of Cre Reporter Expression in a 43 Week Old Femur after Tamoxifen Treatment at E14.5 of Embryogenesis. Cre reporter expressing cells persisted in the bone marrow of 43 week old mice and remain localized toward the proximal end of the femur. (A) Image of femur (distal end – top, proximal end - bottom) showing Cre reporter expressing cells (white). (B–E) Regions of interest along the proximal-distal axis of the femur showing the increase in Cre reporter expressing cells as one moves toward the proximal end of the bone. (TIF) [file pone.0071318.s004.tif]
