# Supplementary material for: Osterix-Cre Labeled Progenitor Cells Contribute to the Formation and Maintenance of the Bone Marrow Stroma
Source: PLoS One. 2013 Aug 8;8(8):e71318. doi: 10.1371/journal.pone.0071318 (PMC3738599; doi:10.1371/journal.pone.0071318)
Supplement: Table S1 — Oligonucleotides used in this study. (DOC) [file pone.0071318.s005.doc]

| **Gene** | **Symbol** | **Sequence** | **Orientation** |
| --- | --- | --- | --- |
| *Osteocalcin* | *Bglap1* | 5'-TCCAAGCAGGAGGGCAATAAG-3' | Forward |
|  |  | 5'-GCGTTTGTAGGCGGTCTTCAAG-3' | Reverse |
| *Bone Sialoprotein* | *IBSP* | 5'-CGCCACACTTTCCACACTCTC-3' | Forward |
|  |  | 5'-CTTCCTCGTCGCTTTCCTTCAC-3' | Reverse |
| *Dentin Matrix Protein 1* | *DMP1* | 5'-CGCATCCCAATATGAAGACTG-3' | Forward |
|  |  | 5'-GCTTGACTTTCTTCTGATGACTCA-3' | Reverse |
| *Osterix* | *Sp7* | 5'-GAGGAGTCCATTGGTGCTTGAGA-3' | Forward |
|  |  | 5'-GGATGGCGTCCTCTCTGCTTGAG-3' | Reverse |
| *Adiponectin* | *Adipoq* | 5'-CGTGATGGCAGAGATGGCACT-3' | Forward |
|  |  | 5'-GCGAATGGGTACATTGGGAACAG-3' | Reverse |
| *Perilipin* | *Plin* | 5'-AGATCCCGGCTCTTCAATACC-3' | Forward |
|  |  | 5'-AGAACCTTGTCAGAGGTGCTT-3' | Reverse |
| *Fatty Acid Binding Protein 4* | *Fabp4* | 5'-GGGAACCTGGAAGCTTGTCTC-3' | Forward |
|  |  | 5'-CTGCGGTGATTTCATCGAATTCCAC-3' | Reverse |
| *Adipsin* | *Cfd* | 5'-GCTATCCCAGAATGCCTCGTT-3' | Forward |
|  |  | 5’-GCGCAGATTGCAGGTTGTC-3’ | Reverse |
| *SRY-box containing gene 9* | *Sox9* | 5-GAGCCGGATCTGAAGAGGGA-3' | Forward |
|  |  | 5'-GCTTGACGTGTGGCTTGTTC-3' | Reverse |
| *Aggrecan* | *Acan* | 5'-CTATGAGGATGGCTTCCACCAGT-3' | Forward |
|  |  | 5'-CCATCTCCTCAGCGAAGCAGT-3' | Reverse |
| *Collagen type 2 alpha 1* | *Col2a1* | 5'-TCGCACTTGCCAAGACCTGAA-3' | Forward |
|  |  | 5'-GGTCTCTCCAAACCAGATGTG-3' | Reverse |
| *Collagen type 10 alpha 1* | *Col10a1* | 5'-GCTGCCTCAAATACCCTTTCTG-3' | Forward |
|  |  | 5'-GGACCAGGAATGCCTTGTTCT-3' | Reverse |
| *Angiopoietin 1* | Angpt1 | 5'-CTCGTCAGACATTCATCATCCAG-3' | Forward |
|  |  | 5'-CACCTTCTTTAGTGCAAAGGCT-3' | Reverse |
| *Chemokine CXC Ligand 12* | *Cxcl12* | 5'-CCAGAGCCAAGCTCAAGCAT-3' | Forward |
|  |  | 5'-CAGCCGTGCAACAATCTGAA-3' | Reverse |
| *Stem Cell Factor* | *SCF* | 5'-GGTAGCTAGTTCTATCCATGCGGT-3' | Forward |
|  |  | 5'-CCTGTAAGGACTTTTCTGGAGAGTCT-3' | Reverse |
| *Glyceraldehyde 3-phosphate dehyrogenase* | *GAPDH* | 5'-AGGTCGGTGTGAACGGATTTG-3' | Forward |
|  |  | 5'-TGTAGACCATGTAGTTGAGGTCA-3' | Reverse |
| *Hypoxanthine guanine phosphoribosyltransferase* | *HPRT* | 5'-CACAGGACTAGAACACCTGC-3' | Forward |
|  |  | 5'-GCTGGTGAAAAGGACCTCT-3' | Reverse |
